# The Functional and Physicochemical Properties of Rice Protein Concentrate Subjected to Acetylation

**DOI:** 10.3390/molecules28020770

**Published:** 2023-01-12

**Authors:** Joanna Miedzianka, Katarzyna Walkowiak, Magdalena Zielińska-Dawidziak, Aleksandra Zambrowicz, Szymon Wolny, Agnieszka Kita

**Affiliations:** 1 Department of Food Storage and Technology, Wroclaw University of Environmental and Life Sciences, 51-630 Wrocław, Poland; 2 Department of Physics and Biophysics, Poznań University of Life Sciences, 60-637 Poznan, Poland; 3 Department of Biochemistry and Food Analysis, Poznań University of Life Sciences, 60-623 Poznań, Poland; 4 Department of Functional Products Development, Wroclaw University of Environmental and Life Sciences, 51-630 Wrocław, Poland

**Keywords:** commercial rice protein preparation, acetic anhydride, amino acid composition, digestibility, FT-IR spectroscopy, functional properties

## Abstract

The aim of the present study was to increase the value of rice protein concentrate (RPC) by improving the functional properties of a preparation subjected to acetylation and analyze the impact of this chemical modification on chemical composition, digestibility, and protein patterning using SDS-PAGE electrophoresis and FT-IR spectroscopy. In the modified samples, the protein content increased (80.90–83.10 g/100 g cf. 74.20 g/100 g in the control). Electrophoresis revealed that the content of the main rice protein fractions (prolamin and glutelin) decreased as the concentration of the modifying reagent increased. Through spectroscopic analysis, wavenumbers, corresponding to the presence of proteins or lipids, aromatic systems, and carbohydrates, were observed. The use of acetic anhydride did not change the digestibility of the modified RPC significantly when compared to that of the control sample. The acetylation of the RPC caused a significant increase in its emulsifying properties at pH 8 (1.83–14.74%) and its water-binding capacity but did not have a statistically significant impact on the oil-absorption capacity. There was a slight increase in protein solubility and a decrease in foaming capacity in the modified RPC.

## 1. Introduction

Plant-based proteins are innovative ingredients with fast-growing applications in the food industry. They are sources of important bioactive compounds and ingredients that are used in the production of functional foods [1]. Sustainability, ethical implications, population growth, variety, and the formulation of healthier products are among their main advantages over animal proteins. However, on the other hand, most plant-based protein preparations obtained by aqueous extraction methods are characterized by poor aqueous solubility, weak functionality (i.e., gelling, emulsification, and foaming), and a high degree of complexity, as well as susceptibility to pH, ionic strength, and temperature, which limits their applications in the industrial sector [2]. Moreover, they are less digestible and have less ability to transport other important nutrients, such as calcium and iron. Therefore, efficient modification processes are needed to improve the value of plant-based proteins. 

One of the methods of protein extraction from plant-based materials that retain their original functional properties is the dry tribo-electrostatic separation method. This technology relies on the efficiency of milling to mechanically dissociate the proteins, which are subsequently separated by an air stream based on particle size and density [3,4]. As a result, the dry fractionation method has many advantages, including having no chemical residues, a minimal impact on the techno-functional properties, and the loss of insoluble protein, as well as having low energy consumption. The low protein extraction yield is the main disadvantage [3]. Based on the studies of Tabtabaei et al. [5], dry tribo-electrostatic separation can produce protein-rich plant fractions from navy bean flour which were characterized by superior solubility, emulsifying, and foaming when compared to wet process isolates. Vitelli et al. [6] investigated the performance of electrostatic separation to produce protein- and carbohydrate-rich fractions from hammer-milled navy bean flour. The results revealed that the samples collected from the middle and top part of the plate had a significantly higher protein content and, therefore, a lower starch content than the samples collected from the bottom of the plate. 

Some of the functional properties can also be changed, inter alia, by chemical modifications [7]. Chemically modified proteins are produced by the addition of new functional moieties or the elimination of the components from the protein’s structure. In proteins, the most reactive nucleophilic groups are the ɛ-amino groups of lysyl residues and the SH group of cysteine. Therefore, these amino acids undergo the most modification. Examples of chemical modification include acetylation and succinylation based on the use of acetic or succinic anhydride, respectively, as the acylating agent. Acylation is a type of modification that has been widely used due to its efficiency, low cost, and ease of operation. Hydrophilic functional groups, such as hydroxyls, ε-amino groups, and phenols, are susceptible to acetylation [8]. Acetylation is advantageous from a technological point of view because the protein concentrates and isolates obtained can have improved solubility, emulsifying ability, and foaming and water-binding capacity and also stabilize the emulsions and determine their color. Additionally, it has been scientifically proven that modification through acetylation may cause changes in the chemical composition of the obtained preparations without adversely affecting their nutritional value [8,9,10]. However, the chemical modification methods (including acetylation with acetic anhydride) are not favorable for food applications as they use chemicals and produce chemical byproducts. Additionally, the modified proteins can be less digestible and are not utilized in animal feeding tests.

One of the plant-based alternatives to meat or soy protein is rice (*Oryza sativa* L.). It is a staple cereal and is widely consumed around the world. It has high nutritional value, being a source of starch, mineral salts, vitamins, and dietary fiber. The protein content of rice is relatively small (about 3%). However, rice proteins have been recognized as highly nutritious, hypoallergenic, and particularly healthful for human consumption. Additionally, the antioxidant and nutraceutical properties of rice are associated with a reduction in the risk of oxidative stress, which contributes to the prevention of hypercholesterolaemia [11,12]. 

Commercial rice protein concentrate is first subjected to alkaline extraction, and then the proteins are precipitated by adjusting the pH to their isoelectric point, where the nonprotein components are isolated by enzymatic processes [12,13]. Depending on factors such as the rice cultivar and the degree of milling of the rice, the protein content of the products of these treatments ranges from 65% to 90%. Rice protein concentrate (RPC) produced on an industrial scale is popular in the food and pharmaceutical industries, mainly in protein supplements and also in drink shakes, bars, or gels. Additionally, studies indicate that RPC can be used as a value-added ingredient in the production of bread [14] or biscuits [15]. It is considered as a substitute for soy protein as it has no beany flavor [16]. However, its powder presents fine particles and poor dissolution properties, limiting its use. 

So far, rice protein has been subjected to chemical modification by phosphorylation with sodium trimetaphosphate (STMP) [17,18] and alkaline deamidation [19]. As for acetylation using acetic acid anhydride, authors have analyzed the physicochemical and functional properties of rice bran protein concentrate [17] and rice protein successively subjected to trypsin hydrolysis [20]. The novelty of our research is the analysis of commercial RPC, and it is known that among the factors which have a great influence on the properties of modified plant protein preparations are the protein’s origin and method of isolation, as well as the conditions of the modification process [21]. The poor functional properties of RPC limit its use, but due to the high biological value of these proteins, it is justified to search for modification methods that might lead to the improvement of its functional properties. Therefore, the aim of this work was to try to improve the functional properties of commercial RPC by acetylation using acetic anhydride and to analyze the influence of the modification on the physicochemical properties (chemical composition, structure, protein profile, and digestibility).

## 2. Results and Discussion

### 2.1. Functional Properties

#### 2.1.1. Protein Solubility Index (PSI)

**Figure 1:**
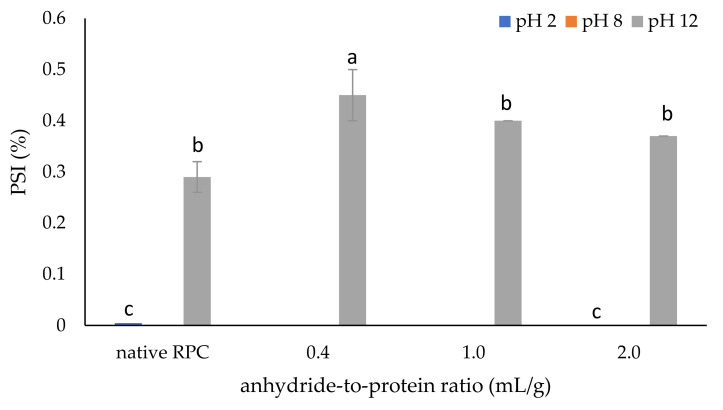
Effect of anhydride-to-protein ratio on the protein solubility index (PSI) of native and acetylated RPC. a, b, c—the same letters within the same analysis indicate values that are not significantly different; RPC—rice protein concentrate; 0.4, 1.0, 2.0—rice protein preparations after acetylation conducted with different concentrations of acetic anhydride (mL/g).

Solubility is often used as an indicator of other functional properties. It is defined as the ability of a substance to form homogeneous mixtures with other substances. The solubility of the control and modified proteins in this study was pH-dependent, as indicated in Figure 1. Over the pH range studied [2,12,16], both the unmodified and acetylated RPC were soluble almost exclusively at pH 12; however, even under this condition, the solubility was very low (below 0.5%). The acetylation of the RPC at pH 12 with a 0.4 mL/g dose of acetic anhydride caused an almost two-fold increase in PSI to 0.45%, as compared to the control sample (0.28%). This could have resulted from the covalent incorporation of the acetyl groups into the amino group of the protein, which results in a certain degree of protein unfolding (electrostatic attraction is diminished). The hydrophilic groups can be exposed, and therefore hydrophilicity increases, which improves solubility [17]. 

As the dose of acetic anhydride increased (from 1.0 mL/g (with a degree of acetylation of 92.24%) to 2.0 mL/g (with a degree of acetylation of 99.98%), data presented in Table 1), the protein solubility decreased, which is in line with the reports by other authors [8,22,23]. The data provided by Heredia-Leza et al. [21] show that when the degree of acetylation does not exceed 78%, the solubility of proteins increases. Thus, the solubility depends on the degree of acetylation, and, in some cases, a deep degree of acetylation is not desired. Similar results have been found for pumpkin proteins wherein a dose of 2 mL of acetic anhydride/g protein caused a decrease in the solubility from 62.44% to 49.52% [21]. Moreover, hydrogen bonds play a fundamental role in regulating protein and water interactions and rely on polarity. RPC is characterized by low polarity; thus, it cannot interact and form hydrogen bonds; therefore, the solubility of the control and acetylated samples was very low [21]. 

In an alkaline environment, protein molecules may unfold, increasing the exposure of the hydrophobic groups and thus changing the solubility of the protein. However, considering the low solubility of the RPC and acetylated samples, this change may have resulted from the increased degradation of the commercial protein, which resulted in fewer interactions between the protein molecules. Additionally, the high insolubility of rice protein in water could be due to extensive aggregation and crosslinking through the disulphides of one of the main fractions, glutelin [8]. Rice proteins are composed of two major hydrophobic fractions: glutelins, which are high-molecular-weight proteins composed of subunits bound by disulphide linkages and are soluble only in dilute acid or alkali, and prolamins, which are soluble in 70% ethanol [11,13]. Also, according to the results of these studies [11,13], RPC displays poor solubility in the pH range of 2–10, with a minimum at pH 5. When considering the amino acid profile (Table S2), it also can be stated that rice proteins have an acidic character; that is, the sum of the aspartic acid and glutamic acid residues (25.95 g/100 g protein) is greater than the sum of the lysine, arginine, and histidine residues (12.51 g/100 g protein). Therefore, they exhibit maximum solubility at alkaline pH. Additionally, the very low solubility of the RPC samples can be explained by the protein isolation method used and, therefore, by the enhanced hydrophobic interactions [10]. Generally, protein preparations with low solubility in water can potentially be applied to products, e.g., meat analogs, baked goods, breakfast cereals, protein bars, and pet food.

#### 2.1.2. Water- and Oil-Absorption Capacity

**Figure 2:**
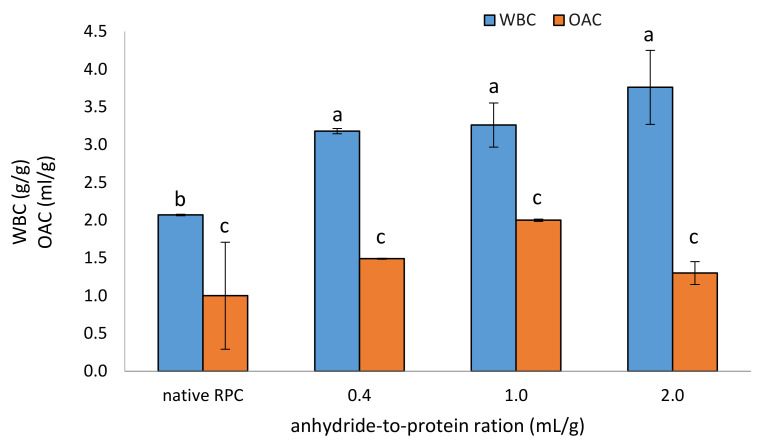
Effect of anhydride-to-protein ratio on water-binding capacity (WBC) and oil-absorption capacity (OAC) of native and acetylated RPC. a, b—the same letters within the WBC analysis indicate values that are not significantly different; c—the same letter within the OAC analysis indicates values that are not significantly different; RPC—rice protein concentrate; 0.4, 1.0, 2.0—rice protein preparations after acetylation conducted with different concentrations of acetic anhydride (mL/g).

Water-binding capacity (WBC), defined as the ability of a protein to retain water in a physical or physicochemical way regardless of the forces of gravity or heating, is a common property of all proteins and protein products. This property is dependent on amino acid composition, hydrophobicity, pH, temperature, and ionic strength [24]. The results indicate that WBC increased after acetylation, already at the dose of 0.4 mL/g and almost two-fold at the dose of 2.0 mL/g (3.76 g/g), as compared to the control rice protein (2.07 mL/g) (Figure 2). No statistically significant difference was found between the acetylated samples. However, we noted that, contrary to the trend observed for solubility, that WBC increased slightly as the degree of acetylation increased. This is in line with the results of Lawal [25], who also observed that water absorption capacity increases with an increase in the level of modification. In his study on African locust bean protein isolate, the highest water absorption capacity of 6.20 mL/g was recorded with 73.6% acetylation. WBC increases after acetylation because, during the reaction, the hydrophobic properties of the control protein are reduced after the modification through the incorporation of additional hydrophilic groups from acetic anhydride. After chemical modification, the high-molecular-weight protein dissociates, which facilitates enhanced water absorption, which is also observed. Similar results have also been noted for jack bean protein [22], African bean protein isolate [25], and acylated mucuna protein [26]. However, the acetylated rice protein showed a low WBC when compared to other plant-based proteins subjected to the same chemical modification. This observation can be explained by differences in the chemical characteristics of the different plant-based protein preparations, such as the total protein content and the changes in their structure resulting from different isolation and modification conditions. The high WBC of a rice protein is related, in part, to its amino acid composition—the greater the number of charged residues (glutamic and aspartic acids), the greater the WBC (Table S2). Such proteins with a high WBC can be used in meat sausages, cakes, or bread. An acetylated preparation of RPC can be used in the processing of meat, fish, and plant products because it increases their juiciness, improves their rheological properties, and reduces weight loss during heating [10].

Oil-absorption capacity (OAC) is the physical entrapment of fat molecules and is influenced by the protein concentration in the preparation, particle size, and porosity, availability of hydrophobic amino acid groups, and protein–fat–carbohydrate interactions. This functional property improves the mouthfeel and flavor retention of certain food products [12]. The control RPC sample was characterized by a low OAC (amounted 1.0 mL/g), which could be related to its high density and particle size (Figure 2). The acetylation using 1.0 mL/g of acetic anhydride resulted in a two-fold increase in the OAC of the modified RPC. In contrast to the WBC, a decrease in the ability to absorb oil was observed as the degree of acetylation increased. The same results were obtained by Lawal [25]. In his study, OAC reduced progressively with an increase in the level of modification, and the lowest OAC of 0.80 mL/g was recorded for African locust bean protein isolate, with 73.6% acetylation compared with the 1.90 mL/g recorded for the unmodified protein isolate. This could be related to the increased net negative charge and consequent decrease in hydrophilicity of the control protein after acetylation.

#### 2.1.3. Foaming Properties

**Figure 3:**
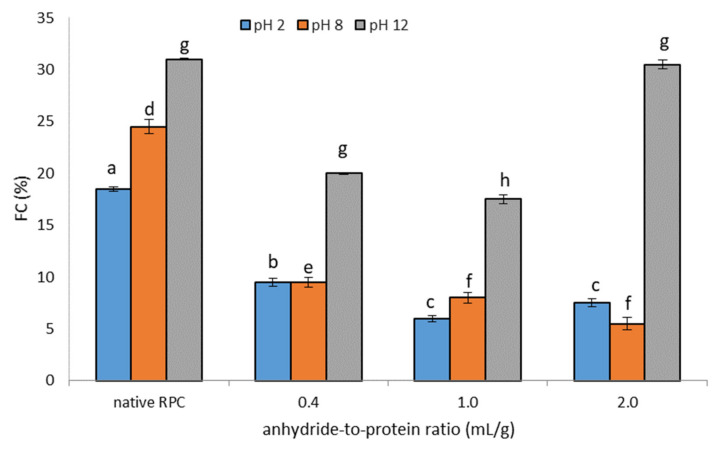
Effect of anhydride-to-protein ratio on foam capacity (FC) at different pH of native and acetylated RPC. a, b, c—the same letters within the pH 2 indicate values that are not significantly different; d, e, f—the same letters within the pH 8 indicate values that are not significantly different; g, h—the same letters within the pH 12 indicate values that are not significantly different. PRC—rice protein concentrate; 0.4, 1.0, 2.0—rice protein preparations after acetylation conducted with different concentrations of acetic anhydride (mL/g).

The ability of a compound to create a foam is called the foaming capacity (FC). Foam is a dispersion mixture in which the dispersing medium is a liquid or a solid, and the dispersed phase is a gas [21]. The effect of acetylation on the FC measured at different pH values is presented in Figure 3. Generally, the acetylation reaction led to a deterioration in the foaming measured at different pHs. However, as the dose of the modifying reagent increased (from 0.4 to 2.0 mL/g), there was a decrease in FC under the same conditions, except at pH 12 for the 2.0 mL/g dose, where the FC was comparable to that of the FC of the unmodified RPC. Similar observations were noted for the foam stability (FS) of rice preparations subjected to modification by acetylation (Table S1). A decrease in the FC of plant-based protein preparations subjected to acetylation with an increasing degree of acetylation was also observed by Bora [27] and Miedzianka et al. [28]. Furthermore, from the review prepared by Heredia-Leza et al. [21], it can be concluded that an increase in the FC of different plant-based proteins can be obtained up to a certain level of acetylation. For example, the 26% acetylation of canola protein leads to a 4.38-times increase in FC, while for 62.5% acetylated lentil and 78% acetylated Bambara groundnut, the FC does not increase significantly [21]. In the studies presented here, RPC acetylation ranged from 71.23% to 99.88%, which could even cause the deterioration in FC. The results disagree with previous observations by El-Adawy [8] and Bora [27]. They noted that the acetylation of canola, mung bean and lentil protein samples, respectively, improves FC because this reaction introduces acetyl groups for the ε-amino groups, lowering the number of positive charges. Additionally, the molecular size of acetylated proteins diminishes, which allows them to move faster toward the air–water interface [21]. A low FC can also be related to low protein solubility. Acetylated RPC, characterized by a low FC, cannot be used in the production of, for example, ice cream. 

#### 2.1.4. Emulsifying Properties

**Figure 4:**
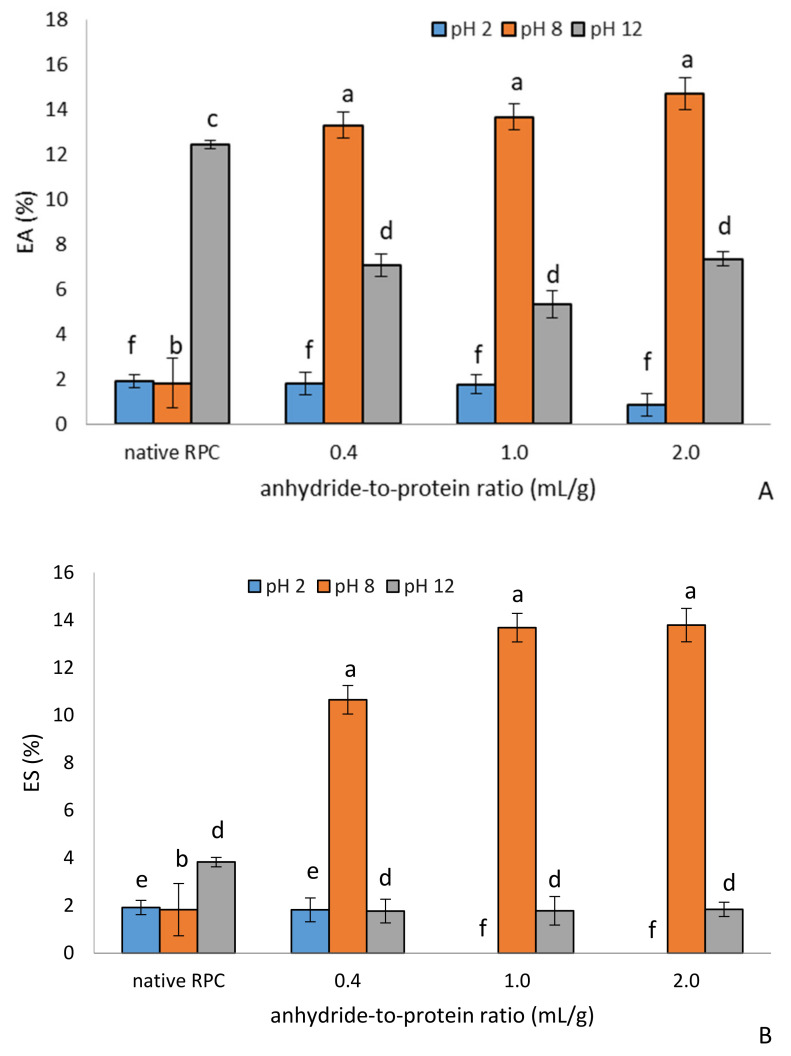
Effect of anhydride-to-protein ratio on emulsion activity, EA (**A**), and emulsion stability, ES (**B**), at different pH for the native and acetylated RPC. a, b—the same letters within pH 8 indicate values that are not significantly different; c, d—the same letters within pH 12 indicate values that are not significantly different; e, f—the same letters within pH 2 indicate values that are not significantly different; RPC—rice protein concentrate; 0.4, 1.0, 2.0—rice protein preparations after acetylation conducted with different concentrations of acetic anhydride (mL/g).

Emulsifying properties are among the most important properties in the manufacturing of many formulated foods. Emulsifying activity (EA) is defined as the ability of a protein solution or suspension to emulsify oil, whereas emulsion stability (ES) is the ability to remain unchanged over a period of time at a specific temperature and gravitational force [12,21]. The type of protein, its concentration, and pH are factors that influence emulsifying capacity [21]. The EA and ES of the control and acetylated RPC were strongly pH-dependent, as indicated in Figure 4A,B, respectively. The acetylation reaction had a significant influence on the EA of the commercial rice sample at pH 8.0. At a dose of 1.0 mL/g and 2 mL of acetic anhydride/g, the EA increased by 7.31 and 8.03 times, respectively. Here, under this condition, the modification enhanced the protein–oil interaction and thus the exposure of the hydrophobic and lipophobic residues, which results in better emulsifying properties. Furthermore, according to [13], the EA of rice protein isolate was relatively low, especially at pH < 6. As observed in Figure 4A, the EA of RPC acetylated with the lowest dose of acetic anhydride improved by more than seven times compared to its control counterpart. This result agrees with previous reports on mung bean isolate [8] and African locust bean [25]. At pH 2 and 12, a decrease was observed in EA and ES with an increasing dose of acetic anhydride. A minimal EA of the control and acetylated RPC was observed at pH 2 due to the limited solubility in this isoelectric region. These acetylated preparations, characterized by strong emulsifying properties (pH 8), may be of importance in the production of finely ground meat products, coffee whiteners, milk, or frozen desserts.

### 2.2. Chemical Composition

The characteristics of control and acetylated RPC are presented in Table 1. The analyzed samples differed statistically significantly in their protein, fat, and ash contents. No differences in dry matter content were observed. All of the analyzed samples met the condition that the water content in the protein preparation cannot exceed 10% [28].

The protein content of the nonacetylated RPC reached a value of 74.20 g/100 g, which resulted from the protein content of the rice seeds (approximately 3% of dried seeds). This commercial product was obtained under harsh processing conditions, and it was recovered in a denatured state with poor functional properties. Along with an increase in the acetic anhydride concentration from 0.4 to 2.0 mL/g, the preparations were characterized by a higher protein content than that of the control sample not subjected to chemical modification. However, the protein content in the acetylated samples was not statistically significantly different and ranged from 80.90 to 83.10 g/100 g. However, it can be seen that as the degree of acetylation increased, the protein content decreased. The higher protein content in the acetylated rice samples could be related to a series of operations performed during the preparation of the modifications. Similar results were noted by Khader et al. [29] upon the acetylation of whey protein concentrate. It is worth highlighting that the acetylation of plant-based protein preparations affects their protein content differently. 

According to the data presented in Table 1, the fat content decreased by up to 75% after the acetylation of the protein with a dose of 1.0 mL/g. This could be related to washing during the precipitation of the modified proteins. Similar results have been noted for acetylated pumpkin protein concentrate, with the fat content ranging from an average of 9.17 g/100 g before chemical modification to an average of 8.89 g/100 g in the modified pumpkin protein concentrate [28].

Plant-based protein preparations are characterized by an ash content of less than 10%, which is mainly affected by the type of raw material. The control RPC, not subjected to chemical modification, contained only 2.15 g of ash/100 g. Along with an increase in the anhydride-to-protein-ratio, the ash content decreased, so that at a dose of 2.0 mL/g an almost 28% decrease in the analyzed compound was observed. This may be related to the removal of excess modifying reagent (because the acetylated samples were washed 3–5 times). Similar results have been noted for acetylated jack bean protein [22] and acetylated pumpkin protein concentrate [28]. 

### 2.3. Amino Acid Composition

**Figure 5:**
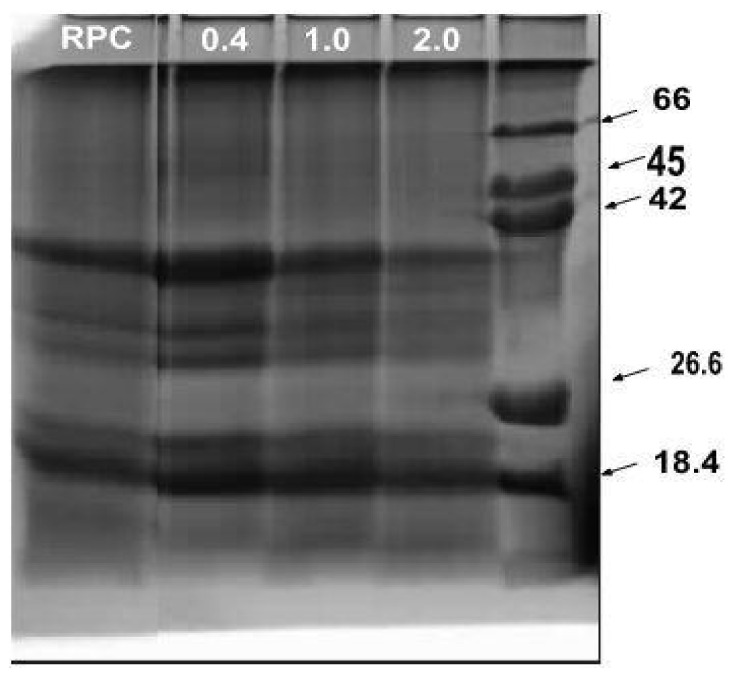
SDS-PAGE of the native and acetylated RPC. RPC—rice protein concentrate; 0.4, 1.0, 2.0—rice protein preparations after acetylation conducted with different concentrations of acetic anhydride (mL/g); MW (kDa)—molecular weight marker prepared in the laboratory (18.4 kDa (β-lactoglobulin), 26.6 kDa (β-casein), 42, 45 kDa (ovalbumin), 66 kDa (bovine serum albumin).

The amino acid composition of a protein significantly affects its functional properties. As presented in Table S2, glutamic acid (17.05 g/100 g protein) was the dominant amino acid among those in the control sample, followed by aspartic acid (8.90 g/100 g protein), valine (7.14 g/100 g protein), and leucine (6.53 g/100 g protein). Proline (1.50 g/100 g protein), histidine (2.21 g/100 g protein), and lysine (3.43 g/100 g protein) were the least noted in the nonacetylated RPC. Other authors have found rice protein to be a source of methionine as well as branched-chain amino acids (BCAA), i.e., leucine, isoleucine, and valine, with lysine as the limiting amino acid. Similar levels of amino acids in a rice protein preparation have been reported by other authors [12,13,16]. The acetylation of the rice protein preparation did not statistically significantly affect the amino acid profile, except for the histidine content, which decreased at higher doses of acetic anhydride (1.0 and 2.0 mL/g). We proved that with an increase in the concentration of the modifying reagent, the content of the main rice protein fractions, i.e., prolamin and glutelin, decreases (Figure 5). Most likely, the change in the protein profile slightly changed the histidine content.

### 2.4. Degree of Acetylation and In Vitro Digestibility

In the analyzed samples, an increase in the degree of N-acetylation was observed along with an increase in the concentration of acetic acid anhydride (Table 1), but only when using the highest dose of acetic anhydride (2.0 mL/g), resulting in an almost complete blockage of the amino acid residues observed (99.88%). This means that there was a decrease in the number of ε-amino groups involved in the acetylation reaction, leading to an increase in the degree of N-acetylation. The degree of acetylation after the chemical modification of the rice protein was higher than that of other plant preparations, which confirms that the source of the protein and the conditions of its acetylation have the greatest influence on them. Some modified plant-based preparations reach only 70–80% acetylation [28]. 

The amount of protein ingested by an organism in proportion to the amount consumed is called digestibility, and this depends on the structure of the protein, the pre-processing, and the antinutritional compounds present [21]. Following the digestion of the analyzed commercial and acetylated rice preparations, the determined protein digestibility ranged from 66.80% (for the samples acetylated with a dose of 1.0 mL/g) up to 70.00% (for the samples not subjected to a modification) (Table 1). Additionally, Amagliani et al. [12] confirmed that the protein digestibility of rice is higher than that of other major cereals (i.e., wheat or corn). The use of acetic anhydride did not change the digestibility of the modified RPC significantly compared to that of the control sample. Similar observations were noted by Bergner et al. [30] and are in accordance with the effect of the process on the amino acid profile discussed above. The influence of the acetylation of lysine on its bioavailability has not been presented in the literature, and thus it is expected that the accessibility of this amino acid to trypsin was not modified. The effectiveness of digestive enzymes (both pepsin and serine proteases) should not be influenced by decreasing histidine content because they do not act on the bond formed with the amino acid [31,32]. On the other hand, acetylation may have a positive effect on the digestibility not related to the protein profile; it may contribute to a significant increase in the digestibility attributed to the destruction of antinutritional factors through chemical modifications [21].

### 2.5. Sodium Dodecyl Sulfate–Polyacrylamide Gel Electrophoresis (SDS-PAGE)

The control and resulting acetylated RPC were analyzed by SDS-PAGE (Figure 5). Based on the electrophoretic analyses, it was revealed that the rice protein not subjected to acetylation was composed of glutelin, with a molecular mass of about 22 kDa and 35–39 kDa, and prolamin, with a molecular mass of about 18 kDa. Similar observations were presented by Wang et al. [11], who additionally observed globulin and proglutelin fractions with molecular masses of 26 kDa and about 57 kDa, respectively. Based on the data in the literature, the major component of rice protein is glutelin, constituting about 80% of the total endosperm protein: prolamin, a minor storage protein, accounts for 5% to 10%. Other proteins include fractions such as globulin and albumin [11]. 

Based on the presented studies, it was shown that with an increase in the concentration of the modifying reagent (from 0.4 to 2.0 mL/g), the content of the main rice protein fractions, i.e., prolamin and glutelin, decreased. The applied acetylation process contributed to the removal of those proteins with a mass of about 18 kDa and about 35 kDa. This also confirmed a decrease in the protein content of the acetylated preparations. Similar results were observed in previous studies [28] when pumpkin protein concentrate was subjected to the same chemical modification. 

### 2.6. Fourier Transform Infrared Spectroscopy (FT-IR)

**Figure 6:**
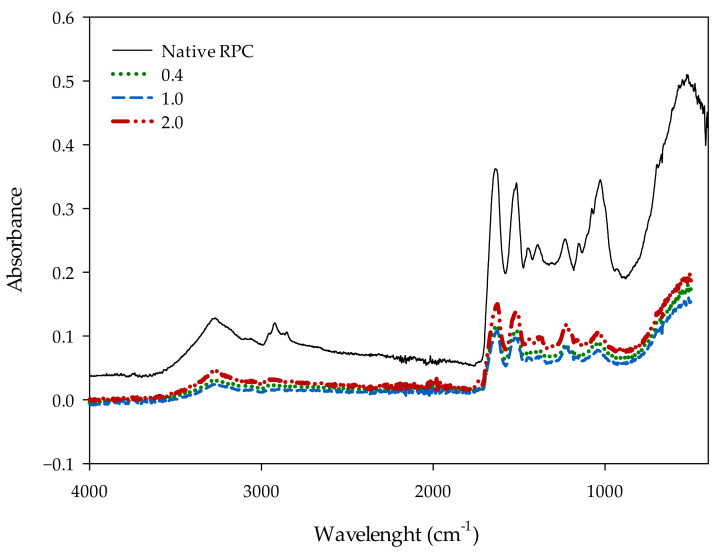
FTIR spectrum of the native and acetylated RPC.

FT-IR is a rapid and noninvasive spectroscopic technique for characterizing various biomaterials. It shows the changes that occur in the tested material at the molecular level, visualized on the basis of the absorption bands of various functional groups present in a given material. By analyzing the spectra of the samples of commercial plant-derived protein concentrate from rice (*Oryza sativa* L.) (RPC) shown in Figure 6, we can observe the maxima at a wavenumber of 1200–1700 cm^−1^, which corresponds to the presence of proteins or lipids. The enhanced absorption bands at wavenumbers of 1630 and 1517 cm^−1^ can be attributed to the presence of the C=C vibrations characteristic of aromatic systems. In all samples, the presence of a region corresponding to carbohydrates (1200–900 cm^−1^) was also apparent [33]. For the first sample, we observed the absorption maximum in the wavenumber area of 2929 cm^−1^, which corresponds to the C-H stretching vibrations. These vibrations did not occur in trials 2, 3, and 4, in which the RPC was acetylated. When analyzing the course of the absorption changes at higher wave numbers, we observed a band at 3300 cm^−1^, which indicates the presence of -O-H- functional groups for each of the tested samples, as well as for C-H and O-H stretching vibrations [34]. 

## 3. Materials and Methods

### 3.1. Materials and Chemicals

Commercial RPC was purchased from the Diet Food company (Opatówek, Poland). The producer recommends direct consumption in the amount of 2–3 tablespoons a day, depending on the body’s needs and physical effort. Acetic anhydride, 2,4,6-trinitrobenzenesulfonic acid (TNBS) were obtained from Sigma-Aldrich (St. Louis, MO, USA). All chemicals used in the experiment were of an analytical grade. 

### 3.2. Acetylation of RPC

Acetylated RPC samples were prepared using the method of Miedzianka et al. [28]. Briefly, the commercial RPC was acetylated with acetic anhydride by adding different concentrations of modifying reagent (0.4, 1.0, and 2.0 mL of anhydride per 1 g of protein contained in the preparation) to the 1% aqueous suspension. After 60 min, the precipitate was centrifuged using an MPW-351 centrifuge (3720× *g* force for 15 min, Heraeus Sepatech, Osteorode, Germany), freeze-dried using Christ Alpha 1–4 LSCplus lyophilizer (Osterode am Hatz, Germany), and then the preparation was sieved and stored at about −20 °C until further analysis. Not subjected to the acetylation process, the native, commercial RPC was used as the control. Acetylation was performed over two technological repetitions. 

### 3.3. Determination of Functional Properties

#### 3.3.1. Effect of pH on Protein Solubility

The pH-solubility profile index (PSI) of the native and acetylated RPC was determined according to the method of Achouri et al. [35] with slight modifications. Briefly, 750 mg of preparation was weighed in the tube, and 15 mL of distilled water was added, and then the pH was adjusted (2, 8 or 12) using either 0.5 M NaOH or 0.5 M HCl. The protein solutions were shaken at room temperature for 30 min and successively centrifuged at 4500× *g* for 15 min (Rotofix 32A by Hettich, Tuttlingen, Germany). The protein content of the supernatants was determined by the Lowry method. Protein solubility was calculated as:

PS = (PCS/TPC) × 100 (%)

where PCS is the protein content in the supernatant after centrifugation and TPC is the total protein content present in the protein sample. 

#### 3.3.2. Water-Binding Capacity and Oil-Absorption Capacity

Water-binding capacity (WBC) of the native and acetylated RPC was determined according to the method described by Jeżowski et al. [36]. For this, 1 g of powdered sample was weighed in a test tube containing 20 mL of distilled water. It was allowed to mix in a laboratory mixer for 60 s and was allowed to stand for 15 min at ambient temperature. This slurry was centrifuged at 4500× *g* for 15 min (Rotofix 32A by Hettich, Tuttlingen, Germany). The separated solid was oven-dried. WBC was expressed as the amount of water (g) absorbed by 1 g of the preparation. 

Oil-absorption capacity (OAC) was determined using the method of Wu et al. [37]. Briefly, 1 g of protein preparation was weighed in the test tube and mixed with 15 mL of rapeseed oil using a Vortex mixer. Samples were allowed to stand for 30 min. The resulting protein–oil mixture was separated using a centrifuge (4000× *g*; Rotofix 32A by Hettich, Tuttlingen, Germany) for 10 min. Immediately after centrifugation, the supernatant was carefully poured into a 15 mL graduated cylinder, and the volumes were recorded. OAC was expressed as the amount of oil (mL) absorbed by 1 g of the preparation. 

#### 3.3.3. Effect of pH on Foaming Capacity and Stability

Foam capacity (FC) and stability (FS) were measured according to the method of Jeżowski et al. [36]. Briefly, 1 g of the preparation was weighed into the tube, and 200 mL of distilled water was added to it. The resulting mixture was adjusted to the appropriate pH (2, 8, or 12) using 0.5 M NaOH or 0.5 HCl. The sample was then homogenized for 2 min at 16,000 rpm (T25 basic ULTRA-TURRAX^®^; IKA Werke, Staufen im Breisgau, Germany). The beaten sample was immediately transferred to a measuring cylinder where the total foam volume was determined after 0, 5, 10, 30, and 60 min. FC and FS were calculated according to the following equations:

FC = (VA/VB) × 100 (%)

where VA denotes the volume after whipping (mL) and VB is the volume before whipping (mL).

FS = (VC/VT) × 100 (%)

where VC denotes the volume before whipping (cm^3^) and VT is the volume after a certain time (mL).

#### 3.3.4. Effect of pH on Emulsifying Properties

Emulsification activity (EA) and stability (ES) were estimated by the method described by Miedzianka et al. [28]. Briefly, the protein suspensions with added oil were mixed using a T25 basic ULTRA-TURRAX^®^ homogenizer (IKA-Werke GmbH & Co. KG, Staufen im Breisgau, Germany) to produce a crude emulsion for 1 min at 20,000 rpm. Then, they were centrifuged at 3000× *g* for 10 min. Emulsion stability was determined by centrifugation after heating at a temperature of 80 °C for 30 min.

EA = a/b × 100 (%)

where a is the height of the emulsified layer in the tube and b is the height of the total contents in the tube.

ES = c/d ×100 (%)

where c is the height of emulsified layer after heating and d is the height of the total contents after heating.

### 3.4. Basic Chemical Composition

The moisture and ash content were measured by the constant mass method [38]. The total protein content was calculated from the amount of nitrogen by a 5.95 factor, evaluated according to the Kjeldahl method, using a Büchi Distillation Unit K-355 (Athens, Greece) (https://www.fao.org/3/y5022e/y5022e03.htm#bm3.1 (accessed on 20 April 2022)). Fat content was determined according to the standard method [38]. 

### 3.5. Amino Acid Composition

The amino acid composition of the native and acetylated RPC was determined by ion-exchange chromatography after 23 h of hydrolysis with 6 N HCl at 110 °C, according to the method described previously [28]. The hydrolyzed amino acids were determined using an AAA-400 analyzer (INGOS, Prague, Czech Republic). A photometric detector was used, working at two wavelengths: 440 nm and 570 nm. A column of 350 × 3.7 mm, packed with ion exchanger Ostion LG ANB (INGOS) was utilized. Column temperature was kept at 60–74 °C and the detector at 121 °C. The prepared samples were analyzed using the ninhydrine method. No analysis of tryptophan was carried out.

### 3.6. Measurement of Degree of N-Acylation

The measurement of the degree of N-acylation was prepared, as described by Habeeb [39]. Briefly, to the protein suspensions, 1 cm^3^ of 4% NaHCO_3_ solution and 1 cm^3^ of 0.1% trinitrobenzenesulfonic acid (TNBS) solution were added. After heating at 60 °C for 2 h, the samples were cooled down to room temperature. Next, 1 cm^3^ of 10% SDS and 0.5 cm^3^ of 0.1 N HCl were added. The absorbance of the solutions was read at 335 nm in a Rayleigh UV-2601 PC spectrophotometer (Beijing, China) against a reagent blank. The absorbance of the control protein concentrate was set equal to 100% free amino groups, and the extent of acetylation of the modified samples was calculated based on the decrease in absorbance because fewer amino groups were able to react with the TNBS reagent.

### 3.7. Digestibility of Protein Preparations

The protein digestibility of the native RPC and its acetylated preparation was determined using a method that simulates two-stage digestion [40], omitting the oral cavity and large intestine steps, as these are irrelevant to protein digestion. Gastric digestion was carried out at 37 °C for 2 h. About 0.5 g of the sample was introduced into the water, and the stomach acidic environment was achieved by decreasing the pH down to 2.0 by 1 N HCl and pepsin addition (60,000 U). The first step was stopped by increasing the pH up to 7.4 by 0.1 M NaHCO_3_. The mixture was enriched in bile salts (0.03 g) and porcine pancreatin (0.005 g), which contains the proteases (trypsin, protease A, ribonuclease)amylase and lipase. The intestine digestion was performed in the same conditions (37 °C; 2 h). The digested sample was centrifuged. The remaining proteins present in the supernatant—not digested but extracted from the sample—were removed by trichloroacetic acid precipitation. In the prepared supernatant, with the use of the Kjeldahl method, protein nitrogen was determined [38] and was related in percentage to the amount of protein nitrogen content in the nondigested sample.

### 3.8. Sodium Dodecyl Sulfate–Polyacrylamide Gel Electrophoresis (SDS–PAGE)

SDS–PAGE analysis was performed according to the method of Laemmli [41]. The protein samples were diluted (10 mg/mL) with the buffer containing SDS and β-mercaptoethanol as the reducing reagents and were denatured. Then, the samples were loaded (10 µL) onto gel slabs (12%). At the end of the analysis, the gel slabs were stained with Coomassie Brilliant G-250 dye. Protein molecular weights were analyzed using the Infinity Capt (BioRad) program for electropherogram analysis.

### 3.9. Fourier Transform Infrared (FT-IR) Spectroscopy

FTIR spectra were obtained by a spectrophotometer from the Perkin Elmer company (Waltham, MA, USA) equipped with an ATR device with diamond as the internal reflection element [42]. Data were collected over a spectral range of 4000–800 cm^−1^.

### 3.10. Statistical Analysis

Statistical analysis of all data was performed using one-way analysis of variance (ANOVA). The analysis of the chemical composition and functional properties were performed in duplicate and in triplicate, respectively. Duncan’s range test was used to determine the differences among the samples with a probability level of 0.05. Statistical analysis and standard deviations were determined using Statistica v. 13.3 software (Dell Software Inc., Round Rock, TX, USA).

## 4. Conclusions

Based on the literature data, the acetylation of rice proteins with acetic anhydride, which was used in the presented work, is one of the most effective chemical methods for improving some functional properties without negatively affecting a change in chemical composition. However, it is still difficult to find an explanation for the relationship between the structure of the modified proteins and their function. The acetylation of a commercial rice protein preparation using a dose of 0.4 mL of acetic anhydride/g of protein improved the WBC and emulsifying properties at pH 8, which extends the possibilities of using the modified rice samples in the processing of meat, fish, and plant products, as well as in finely ground meat products, coffee whiteners, milk, or frozen desserts.

## Figures and Tables

**Table 1:** Characteristics of native and acetylated RPC.

Anhydride-to-Protein Ratio (mL/g)	Dry Matter	Protein	Fat	Ash	Degree of N-Acylation	Amount of Protein Released into the Intestinal Fluid in Two-Step Digestion
	(g/100 g)				%	
native RPC	94.49 ± 0.10 ^a^	74.20 ± 1.15 ^b^	6.32 ± 0.09 ^a^	2.15 ± 0.04 ^a^	-	70.00 ± 6.6 ^a^
0.4	97.32 ± 0.16 ^a^	83.10 ± 0.09 ^a^	4.99 ± 0.26 ^b^	1.93 ± 0.00 ^ab^	71.23 ± 0.15 ^c^	69.00 ± 10.4 ^a^
1.0	96.96 ± 0.54 ^a^	82.30 ± 3.69 ^a^	1.58 ± 0.21 ^c^	1.69 ± 0.14 ^b^	92.24 ± 0.17 ^b^	66.80 ± 1.9 ^a^
2.0	96.16 ± 2.21 ^a^	80.90 ± 0.42 ^a^	1.94 ± 0.31 ^c^	1.56 ± 0.27 ^b^	99.88 ± 0.11 ^a^	67.00 ± 5.8 ^a^

Values are means ± standard deviation; *n* = 3; a, b, c—the same letters within the same column indicate values that are not significantly different; RPC—rice protein concentrate; 0.4, 1.0, 2.0—rice protein preparations after acetylation conducted with different concentrations of acetic anhydride.

## Data Availability

Data are contained within the article and Supplementary Materials.

## Supplementary Materials

The following supporting information can be downloaded at: https://www.mdpi.com/article/10.3390/molecules28020770/s1. Table S1: Amino acid composition, Table S2: Foam stability results.

## Sample Availability

Samples of the compounds are available from the Authors.
