# Supplementary material for: The Functional and Physicochemical Properties of Rice Protein Concentrate Subjected to Acetylation
Source: Molecules. 2023 Jan 12;28(2):770. doi: 10.3390/molecules28020770 (PMC9864533; doi:10.3390/molecules28020770)
Supplement: Supplementary file 1 [file molecules-28-00770-s001.zip › molecules-2062870-supplementary.pdf]

Table S1. Amino acid concentration (g/100 g protein) in native and acetylated PPC.

Amino Acids	Native RPC	Anhydride-to-Protein Ratio (mL/g)		
		0.4	1.0	2.0
IAA *				
Leucine	6.53 ± 0.53 [a]	6.58 ± 0.03 [a]	6.21 ± 0.31 [a]	6.70 ± 0.21 [a]
Isoleucine	3.89 ± 0.04 [a]	3.87 ± 0.02 [a]	8.82 ± 0.02 [a]	4.07 ± 0.19 [a]
Methionine	2.60 ± 0.11 [a]	2.52 ± 0.01 [a]	2.47 ± 0.04 [a]	2.65 ± 0.16 [a]
Cysteine	2.58 ± 0.12 [a]	2.55 ± 0.07 [a]	2.57 ± 0.08 [a]	2.78 ± 0.22 [a]
Phenyloalanine	4.89 ± 0.10 [a]	4.81 ± 0.01 [a]	4.74 ± 0.05 [a]	5.04 ±0.27 [a]
Threonine	3.84 ± 0.18 [a]	3.70 ± 0.00 [a]	3.70 ± 0.05 [a]	3.94 ± 0.22 [a]
Lysine	3.43 ± 0.06 [a]	3.34 ± 0.03 [a]	3.31 ± 0.03 [a]	3.47 ±0.10 [a]
Tyrosine	4.44 ± 0.09 [a]	4.42 ± 0.03 [a]	4.32 ± 0.04 [a]	4.61 ± 0.25 [a]
Valine	7.14 ± 0.02 [a]	7.12 ± 0.04 [a]	7.08 ± 0.07 [a]	7.47 ± 0.34 [a]
DAA **				
Aspartic acid	8.90 ± 0.24 [a]	8.72 ± 0.04 [a]	8.53 ± 0.08 [a]	9.06 ± 0.45 [a]
Glutamic acid	17.05 ± 1.36 [a]	17.73 ± 0.18 [a]	15.87 ± 0.67 [a]	16.32 ± 0.14 [a]
Serine	5.45 ± 0.31 [a]	5.27 ± 0.04 [a]	5.17 ± 0.02 [a]	5.48 ± 0.28 [a]
Glycine	4.57 ± 0.22 [a]	4.53 ± 0.01 [a]	4.39 ± 0.03 [a]	4.65 ± 0.18 [a]
Alanine	6.14 ± 0.32 [a]	6.05 ± 0.02 [a]	5.81 ± 0.16 [a]	6.25 ± 0.23 [a]
Histidine	2.21 ± 0.09 [a]	2.18 ± 0.02 [a]	2.12 ± 0.07 [b]	2.14 ± 0.09 [b]
Arginine	6.87 ± 0.17 [a]	6.79 ± 0.01 [a]	6.66 ± 0.01 [a]	7.04 ± 0.37 [a]
Proline	1.50 ± 0.11 [a]	1.91 ± 0.17 [a]	1.40 ± 0.01 [a]	0.85 ± 0.13 [a]
Total amino acids	92.03 ± 3.86 [a]	92.11 ± 0.35 [a]	88.17 ± 0.66 [a]	95.52 ± 3.55 [a]

values are means ± standard deviation; n = 3; [a,b]—the same letter in verse mean homogenous group; IAA *—indispensable amino acids; DAA **—dispensable amino acids.

Table S2. Results of the foam stability (FS, %) at different pH of native and acetylated RPC.

Foam Stability (%)					
**Type of Preparation**		**Time**	**pH**		
			**2**	**8**	**12**
**Native RPC**		5 min	16	21	27.5
		10 min	15.5	16	25.5
		30 min	14.5	6.5	22.5
		60 min	7	5.5	17
**Anhydride-to-Protein Ratio (mL/g)**	**0.4**	5 min	7.5	8.5	18.5
		10 min	6.5	8	10
		30 min	5	5.5	8.5
		60 min	5	4.5	7
	**1.0**	5 min	5.5	6.5	16
		10 min	4.5	6	9.5
		30 min	3.5	5	6.5
		60 min	2.5	0.5	5.5
	**2.0**	5 min	6.5	5	27
		10 min	5.5	3	15
		30 min	5	1	6.5
		60 min	2.5	0	5.5

RPC—rice protein concentrate; 0.4, 1.0, 2.0—rice protein preparations after acetylation conducted with different concentrations of acetic anhydride.
